# Strategies for measuring airway mucus and mucins

**DOI:** 10.1186/s12931-019-1239-z

**Published:** 2019-11-21

**Authors:** Kalina R. Atanasova, Leah R. Reznikov

**Affiliations:** Department of Physiological Sciences, University of Florida, 1333 Center Drive, PO Box 100144, Gainesville, FL 32610 USA

**Keywords:** Airway mucus, Mucociliary transport, Techniques, Mucins

## Abstract

Mucus secretion and mucociliary transport are essential defense mechanisms of the airways. Deviations in mucus composition and secretion can impede mucociliary transport and elicit airway obstruction. As such, mucus abnormalities are hallmark features of many respiratory diseases, including asthma, cystic fibrosis and chronic obstructive pulmonary disease (COPD). Studying mucus composition and its physical properties has therefore been of significant interest both clinically and scientifically. Yet, measuring mucus production, output, composition and transport presents several challenges. Here we summarize and discuss the advantages and limitations of several techniques from five broadly characterized strategies used to measure mucus secretion, composition and mucociliary transport, with an emphasis on the gel-forming mucins. Further, we summarize advances in the field, as well as suggest potential areas of improvement moving forward.

## Background

Increased airway mucus and airway obstruction are hallmark features of many respiratory diseases [1–4]. The composition of mucus and its properties have long been considered informative for airway disease diagnosis and progression. However, studying mucus presents several challenges, including a complex and heterogeneous composition, limitations in collection methods, and laborious procedures for downstream processing. Although, advances in imaging techniques have improved aspects of mucus research, these techniques remain less accessible due to the expertise required and equipment necessary to execute. Here, we review and discuss the advantages and limitations of several techniques from four broadly characterized strategies used to measure mucus properties and mucociliary transport (MCT). The advantages and limitations of such techniques have rarely been discussed. Doing so has the potential to both impact and inform researchers and clinicians alike, which may ultimately influence patient treatment and care.

## Airway surface liquid (ASL) in health and disease

**Fig. 1:**
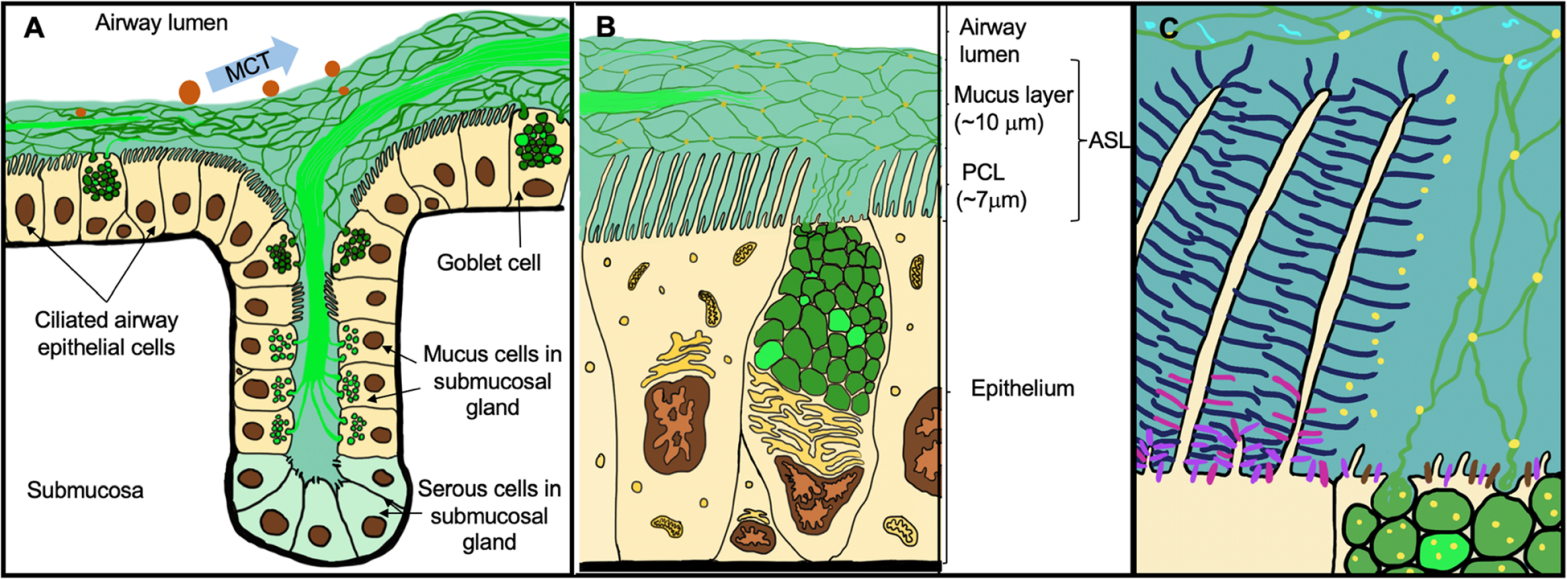
Airway Surface Liquid (ASL) and Localization of the Major Mucins in Healthy Airways. **a** General schematic representation of mucus secretion from goblet cells and submucosal glands. The proposed structure of MUC5AC (threads in dark green) and MUC5B (bundles/strands in bright green) is shown. Mucociliary transport (MCT) of inhaled pathogens and particles (orange spheres of different sizes) is shown with a blue arrow. **b** Schematic representation of the generally-accepted structure of ASL. The periciliary layer (PCL) is estimated to be ~ 7 μm thick under normal conditions. Mucus layer thickness varies among individuals and in the different parts of the airway of the same individual (up to 70 μm) under normal conditions. **c** ASL gel-on-brush model with localization of large airway mucosal epithelium-expressed membrane-tethered mucins (MUC1 = purple, MUC4 = dark blue and MUC13 = pink and MUC16 = brown) and their interactions with secreted gel-forming (MUC5AC = dark green, MUC5B = bright green) and monomeric mucins (MUC7 = light blue; only depicted as incorporated in the gel-layer). Globular, non-mucin proteins that are secreted by different cells and incorporated within the gel mesh are represented in yellow dots in **b**) and **c**). MUC8 and MUC19 are omitted due to the sparsity of data on their secretion and localization in normal respiratory tissues. The MUC2 gel-forming mucin has also been omitted in this figure due to the very low levels of expression and secretion in normal airways (see text for references). MUC20, MUC21 and MUC22 were also omitted

Airway surface liquid (ASL) is the thin liquid film that covers the airways [5]. It protects the airways from desiccation and facilitates the swift removal of inhaled particulates, debris, pathogens and toxicants through mucociliary transport (MCT). From a structural standpoint, ASL consists of two main layers: 1) the apical layer consisting of a water-based polymeric mucus; and 2) a periciliary layer (PCL), also referred to as a sol layer, [6] that bathes the epithelium (Fig. 1). Historically, studies suggest that goblet cells, serous cells and submucosal glands contribute to ASL production [7–10]. The recent discovery of the airway ionocyte [11, 12] might also result in a revised understanding of ASL production.

Although the majority of ASL is water [13], large glycoproteins known as mucins [14] make up a significant portion of the proteins in the apical mucus layer. Mucins are encoded by different *muc* genes, after which the proteins are generally named and numbered in the order of discovery [15, 16]. Currently there are 21 mucins identified in humans (denoted with capital letters), 13 of which are found in the respiratory tract [7, 16]. They can be divided into three classes depending on their ability to polymerize, and on whether they are secreted or are cell surface-bound [7]. These three groups include the secreted monomeric mucins (MUC7, MUC8), the secreted polymeric (gel-forming) mucins (MUC2, MUC5AC, MUC5B and MUC19) and non-secreted surface-bound mucins (MUC1, MUC4, MUC13, MUC16, MUC20, MUC21 and MUC22).

Deviations in the composition of ASL, and particularly the mucus layer, are associated with several airway diseases, including asthma, cystic fibrosis and COPD [1–4, 17]. These alterations can be due to enhanced mucin production and secretion, and/or a reduction in water content. For example, in asthma the enhanced production of MUC5AC due to goblet cell hyperplasia, paired with airway remodeling and inflammation, drive airway morbidity and mortality. Indeed, the Severe Asthma Research Program (funded by the NHLBI) found that 58% of people with asthma exhibited airway mucus plugs [1]. The extent of mucus plugging correlated with airflow limitation and worse control of asthma. Similarly, a recent study involving the AREST CF program found that airway mucus plugging was a significant predictive indicator of future lung function [4], and it is well known that people with cystic fibrosis undergoing lung transplantation exhibit profound mucus plugging of the small airways [2]. However, mucus abnormalities in cystic fibrosis are thought to be due to a combination of events, including ASL dehydration [8, 18], altered electrostatic interactions of mucins [13, 19], impaired mucus detachment [20], as well as changes in mucin content [21]. Airway obstruction is also a common feature of COPD [22]. Recent SPIROMICS data suggested that sputum mucin concentrations, including MUC5AC and MUC5B [23], were markers of disease severity in COPD. Although the mechanisms mediating mucin alterations in COPD are still being elucidated, inflammation [24], smoking [25] and acquired ion channel dysfunction [26] are key contributors.

## Gel-forming mucins

Mucins are heterogeneous glycoproteins [15, 27, 28]. The protein backbones have unique multiple amino-acid tandem repeats containing serines and threonines, where oligosaccharides are covalently linked. The backbones represent ~ 20% of the molecular weight, whereas the carbohydrates account for ~ 80% of the weight [7, 29]. The carboxy and amino terminals of the backbones are rich in cysteine, allowing for end-to-end disulfide bonds and subsequent dimerization or multimerization. This multimerization results in a complex hydrated porous molecular network that, together with the other components secreted by airway epithelial cells and submucosal glands, represent the gel basis of airway mucus [27, 29, 30]. Indeed, once released via exocytosis, mucins can expand more than 100 times their dehydrated size [27, 29, 31]. This property is partly why mucins represent such a large portion of the proteins that make up the mucus layer. The structure, biosynthesis, glycosylation and secretion mechanisms of mucins have been extensively studied and reviewed elsewhere [3, 15, 29, 30, 32], and therefore will not be further addressed here.

MUC5AC and MUC5B represent the major secreted gel-forming mucins and are responsible for the viscoelastic and functional properties of mucus in health and disease [33]. Although MUC2 has been shown to be among the major gastro-intestinal mucins, and gestationally associated with airway developing cells [16, 34], it is expressed and secreted in very small quantities in “normal” airway tracheo-bronchial epithelia [35]. To date there is very little data on MUC19. Its expression in the respiratory system has been localized to submucosal glands [36, 37], but there is limited data to inform on its secretion properties [37].

In addition to mucins, inflammatory cells [38] and host defense proteins [39] are often found in mucus. There is significant diagnostic value in examining inflammatory cells and inflammation profiles. For example in asthma, tailoring treatments based upon presence of eosinophils [40] has been an effective strategy to decrease asthma exacerbations [41]. Neutrophils are also commonly found in airways of individuals with severe asthma [42] and cystic fibrosis [43]. A greater number of neutrophils in the lavage fluid of smokers, as well as the sputum of people with COPD, has also been reported [44, 45]. Neutrophils modify mucus properties and increase mucus viscosity through releasing DNA nets [46, 47]. Thus, in instances where mucus is extremely viscous, agents that “thin” mucus, such as mucolytics, might be required for mucus processing [48]. Additional cells found in healthy and diseased lungs include macrophages and lymphocytes, among others [44].

## Challenges in measuring airway mucus and mucins

Despite strong evidence that deviations in mucus abundance and/or composition drive mortality and morbidity in several airway diseases [1–4], measuring mucus and mucins remains a challenge [49, 50]. For example, obtaining mucus in vivo from the trachea, bronchi and bronchioles can be difficult due to limitations in collection methods, as well as the potential for contamination with saliva. Further, accessing the trachea, bronchi, and lungs in humans and animal models to collect mucus is invasive and can be confounded by MCT.

In vitro measurements, on the other hand, might not be an accurate representation of the in vivo environment, as many of the neural, endocrine and immune systems, which regulate mucus secretion and mucin production, are lacking [51]. For example, mucus secretion and MCT are directly regulated by sympathetic and parasympathetic reflexes [52]. Endogenous sex hormones also play role in diseases such as asthma [53]. Additionally, the formation of mucus plugs that can lethally obstruct the airways are difficult to study in a cell culture system where the airway architecture, including luminal spaces, are missing [1, 54].

Although animal models are often used to study respiratory diseases, they show marked differences in their airway anatomy and structure, including abundance of submucosal glands [32, 51, 55, 56]. These anatomical and physiological differences can make extrapolation of research findings to humans difficult. Further, species-dependent differences in the structure of gel forming mucins [57], including differences in amino-acid sequences [58] and sugar side-chains [59], make standard quantitative and semi-quantitative techniques that use antibodies (e.g., western blot, ELISA, etc.) more arduous.

Another challenge in the mucus biology field is the significant influence that environmental factors, such as infections, inflammation, and smoking, have on mucus properties. As mentioned previously, neutrophils increase mucus viscosity by extruding DNA material [46, 47]. Cigarette smoke, on the other hand, can increase the production of MUC5AC [60] and increase neutrophil number. Additionally, infection with *Pseudomonas aeruginosa* increases sialyation of mucins, which facilitates *Pseudomonas aeruginosa* colonization [61]. Combined, these factors can make sampling and downstream processing of mucus unpredictable and tedious. More extensive information related to environmental influences on mucus is provided by Fahy and Dickey [62].

Despite these challenges, several techniques have been developed to assess mucin expression and mucus content. Below we highlight some of these methodologies and comment on their advantages and limitations, with an emphasis on the gel-forming mucins. Though we focus on gel-forming mucins, many of the approaches discussed here are also applicable to studies centered on airway inflammation and inflammatory cells trapped in mucus.

## Techniques for measuring mucus and mucin

The assessment of mucus can occur on many different levels, requiring many different strategies. Here we outline five broad categories and briefly highlight some common methodologies representative of each category. More detailed information regarding advantages and limitations of techniques discussed is provided in the corresponding tables.

### Collection methods (Fig. 2, Table 1)

An ongoing challenge in the mucus biology field is the collection of samples. There are several strategies that can be implemented, each with distinct advantages and limitations. For airway epithelia grown at the air-liquid interface (ALI), a common practice is to perform an apical wash. This approach is fairly simple, and offers an advantage in that samples can be assayed in response to interventions [63, 64]. However standardized procedures need to be practiced, as accumulated mucins might not be removed properly without successive washing [65]. Further, pooling of samples might be required [66], which necessitates a greater sample size. Any studies in which ALI cultures are used to measure mucus secretion and its properties must account for experimental confounds, such as mechanical stimulation and/or unintentional goblet cell discharge [65].

**Fig. 2:**
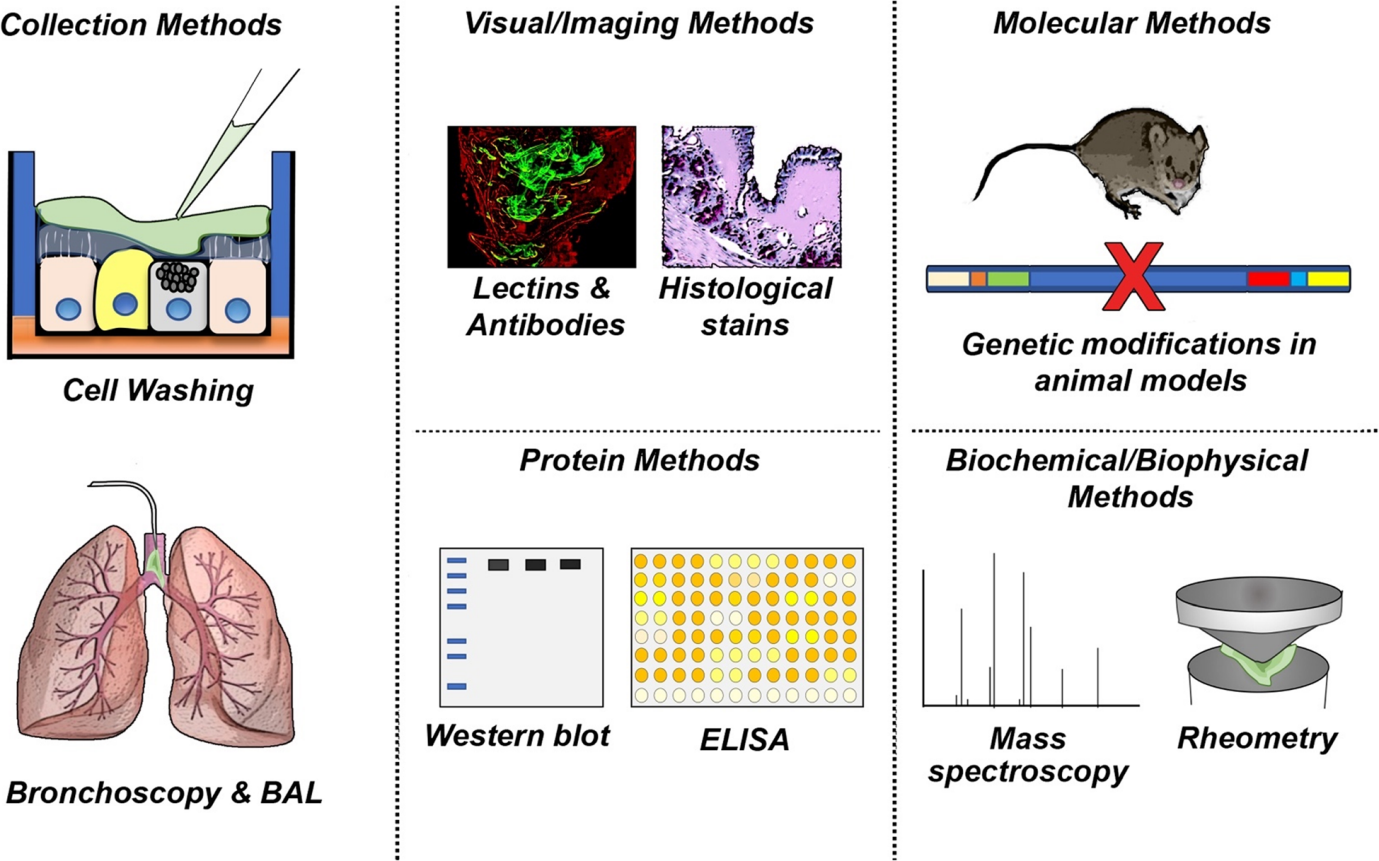
Summary of broadly categorized strategies to study mucus and mucins. General schematic highlighting a few of the methods used to collect and study mucins. Not all methods discussed are shown. Abbreviations: BAL, bronchoalveolar lavage; ELISA, enzyme-linked immunosorbent assay

**Table 1:** Advantages and limitations of mucus and mucin collection methods

Method	Advantages	Limitations
Cell culture wash	- Requires minimal specialized equipment and is not overly tedious. - Can sample mucus in response to interventions [63, 64]. - Potential for repeated collection and/or longitudinal study.	- Accumulated mucins might not be removed properly if washing is not done successively or is incomplete [65]*.* - Samples may require pooling [66].
Bronchoalveolar lavage	- Allows for direct sampling of the airway fluid - Applicable in vivo [67] and ex vivo [68]. - Relatively large volumes can be retrieved. - Materials to perform are standard. - Can be performed in human patients [69]. - Potential for repeated and/or longitudinal sampling.	- Must be clinically indicated in order to perform in humans. - Generally done under local anesthesia in vivo [70]. - Fluid retrieved is a combination of multiple cells and multiple proteins [67]. - Volume recovered is variable [71]. - Non-adherent proteins may be overrepresented
Sputum (spontaneous and induced)	- Provides information about mucus and mucins in the lower airways - Spontaneous sputum requires no intervention for its production - Induced sputum provides a higher proportion of viable cells [72]. - Guidelines in place for inducing sputum in human [73].	- Potential for contaminated with saliva. - Induced sputum usually requires inhalation of hypertonic saline, which can be irritating and change composition of mucus [74, 75]. - Success of sputum induction influenced by inflammation [72]. - Variations in the amount of sputum produced [76]. - Not really applicable to animal models.
Bronchoscopy	- Direct sampling of mucus when used to remove plugs [77]. - Provides significant diagnostic information. - Can be performed in human and animal patients.	- Performance in human patients or animal patients requires highly specialized equipment and training - Typically performed under conscious sedation, occasionally occur under general anesthesia [70].
Endotracheal tube sampling	- Direct sampling of mucus [78, 79].	- Hydration of the mucus varies from the inside or the outside of the tube [80] - Endotracheal tube placement in human and animal patients requires highly specialized training and a licensed medical practitioner.

Another collection approach similar to cell washing is bronchoalveolar lavage (BAL). This technique can be utilized both in vivo [67] or ex vivo [68] and entails the irrigation and retrieval of a known volume of fluid from a defined area of the airway tree. For humans, a BAL requires a bronchoscopy [81]. An advantage of BAL is that it can be utilized in human patients [69] and experimental model systems. However, important limitations are the need for general anesthesia [70], variation in retrieval volumes [71], and the impact that local inflammatory cells can have on the retrieval process (e.g., lung permeability [82]).

Sputum also provides information about mucus and mucins. This heterogeneous material consisting of cells and mucus is expelled from the lower airways via cough. There are two major types of sputum: induced and spontaneous. An advantage of spontaneous sputum is that no clinical intervention is required for its production. Conversely, induced sputum entails aerosolization of hypertonic saline [74, 75] to the airways using standardized protocols [73]. An advantage of induced sputum is that standardized protocols facilitate reproducibility and rigor across studies. Limitations of both spontaneous and induced sputum include the possibility for saliva contamination, as well as variations in the amount of sputum produced [76]. Further, the success of sputum induction is influenced by the degree of inflammation [72] and caution should be practiced when working with asthmatic patients (due to the bronchoconstriction effects of hypertonic saline [83]). Despite these limitations, sputum is widely used to study airway mucus and airway inflammation [84].

Bronchoscopy is also used to collect and study mucus. As highlighted previously, bronchoscopy is required to perform BAL. However, bronchoscopy can also be used to facilitate removal of mucus plugs [77]. In some cases, removal of a mucus plug may necessitate the application of mucolytics [77]. A significant advantage of bronchoscopy is the diagnostic information it provides, as well as the ability to directly sample mucus. Potential limitations (as highlighted above) include the need for general anesthesia and requirement for a licensed medical practitioner to perform.

Lastly, mucus may be collected from endotracheal tubes [78, 79]. Endotracheal tubes are generally placed under two circumstances: critical illness and general anesthesia (for airway management) [85]. Mucus often accumulates in the endotracheal tube [78], and in some cases, creates a plug [86]. Upon extubation, the mucus material can be directly collected and studied from either inside or outside of the tube. An advantage of endotracheal tube mucus sampling is that it is a direct interrogation of airway mucus. However, a limitation of endotracheal tube sampling is that the hydration of the mucus varies from the inside or the outside of the tube [80] which can decrease reproducibility across experiments. Similar to bronchoscopy, endotracheal tube placement in humans requires a licensed medical practitioner.

### Visual and imaging methods (Fig. 2, Table 2)

Several methods, including histological stains (e.g., Alcian Blue (AB), Periodic Acid–Schiff (PAS)), lectins and antibodies remain the most basic and ubiquitously used visual techniques to examine mucus and mucins [90, 92, 93, 100, 101]. These methods offer the advantage in that they are relatively simple to perform. They also offer spatial context, as procedures are typically performed on airway tissue sections. Antibodies offer greater specificity and direct detection of mucins, whereas AB/PAS and lectins are indirect. Important considerations for quantification of histological, lectin and antibody-based methods include maintaining the same fixation [102, 103] and imaging parameters (e.g., lamp or laser intensity, magnification) across samples, imaging a sufficient number of fields to acquire accurate representation of the sample, and ensuring that the samples are acquired from the same anatomical location across subjects [104]. In many cases, collaboration with a pathologist facilitates proper analysis. Additional considerations and recommendations are highlighted elsewhere [105, 106].

**Table 2:** Advantages and limitations of visual and imaging methods and techniques used for measuring mucus properties

Method	Advantages	Limitations
Beads/microspheres visualization and tracking in vitro [87–89]*,* ex vivo [20, 90] and in vivo [91]	- Easily visualized. - Potential for in vivo tracking.	- Most applicable in vitro and in small animal models ex vivo/in situ*.* - Data analysis can require careful application of modelled calculations that present opportunities for error. - Some of the in vivo applications may require expensive visualization set up.
Histology & Immunostaining (using specific antibodies, Lectins, PAS/AB) [90, 92, 93]	- Inexpensive, easily visualized. - Specific antibodies can provide precise mucin detection and localization or co-localization with other molecules. - Fluorescent lectins can be used for semi-quantitation by fluorescence intensity measurement and are inexpensive	- Applicable mostly in vitro and ex vivo. - When scoring systems are utilized, careful analysis by multiple individuals blinded to group treatments are necessary. - Fixation and washing steps might result in mucus being washed away. - Lectins bind to different carbohydrates in the oligosaccharide chains of glycoproteins and glycolipids and therefore are not mucin specific.
Electron microscopy [94]	- In depth view of micro anatomical structures of cells and gel-forming mucins.	- Difficult to detect more than one type of gel mucin at the same time. - The type and duration of fixation is very important for retention of mucin structures.
X-ray imaging analysis [1, 20, 95–98]	- Novel techniques provide in vivo ability to detect mucus - Very recent X-ray synchrotron [96] and quazi-monochromatic X-ray phase-contrast imaging techniques have been applied successfully to measure MCT in vivo, together with lung motion. - Can detect mucus plugs in humans in vivo*.*	- Expensive set-up and materials. - Potential for exposure to harmful rays. - At the moment, are limited in utility for longitudinal in vivo studies. - Highly specialized equipment and skills
Volumetric – submucosal gland bubble visualization [55, 99]	- Detect ex vivo/in vitro increased output from single cell or multiple glands under normal or treatment conditions. - The total volume technique gives a simple quantitation of total mucus secretion ex vivo/in vitro for a constant time period at baseline and/or after treatment.	- Volume output may not necessarily comprise only mucus but can also include changes in serous gland- and non-glandular cell-secretions. - Currently not applicable in vivo.

Mucus thickness, viscoelasticity, and transport properties can be examined in vitro and ex vivo using microscopy with fluorescent probes and dyes that label or diffuse through mucus [20, 87, 88, 91, 95, 107]. Imaging as a tool to measure viscoelastic properties is covered in greater detail in a subsequent section. Experiments in which fluorescent probes and particles are used to assess mucus require careful consideration, as factors such as pH sensitivity, photostability, brightness, and size influence experimental design and outcome. However, an advantage of using fluorescent probes, particles, and dyes is that they offer spatial and temporal resolution when visualized by a microscope. This can be beneficial for investigating experimental or therapeutic interventions, as well as delineation of the layers of ASL impacted by an intervention or disease. Further, these techniques can be used in multiple species, making comparative studies possible. Finally, because these methods are used quite extensively, they are generally well-accepted. However, one limitation is the lack of specificity for studying mucin (e.g., assessing mucus and ASL, not mucin directly). Additionally, because many experiments using fluorescent probes and particles occur in vitro or ex vivo, careful control of environmental conditions, which may necessitate environmental chambers, is needed to minimize the introduction of unintentional artifacts and confounds.

Several imaging techniques have been pioneered over the years that allow for examination of mucus (and ciliary) function in vivo through assessment of MCT. Currently, in humans, measuring MCT in the conducting airways of the lung has been chiefly accomplished through radioactive aerosols [108]. These studies can be laborious and time-consuming and require highly specialized and standardized testing environments/equipment. Further, initial deposition of radioactive aerosols, which is impacted by particle size and breathing, greatly influence the rate by which mucus is cleared [108]. This can create “noise” and increase the sample number required to detect differences within and across human populations.

In animal models, fluorescent or radiopaque particles have been used to measure MCT in vivo and ex vivo. Advanced imaging modalities are often required to conduct these experiments, and the post-hoc analysis can be quite extensive and demanding, depending upon the method used. However, these techniques offer a greater resolution and granularity compared to MCT studies conducted in humans that use radioactive aerosols [20]. They also allow for the impact of interventions on MCT to be assessed in vivo. Additional assays to visualize mucus and mucins include volumetric determination of submucosal gland secretions using an oil interface [55, 99] and electron microscopy [94]. Newer techniques that visualize mucus flow and MCT by scintigraphy [109] and X-ray imaging techniques [1, 20, 96–98] have also been employed. In some cases, these newer techniques allow for the detection of mucus plugs in humans [1].

### Molecular and genetic approaches (Fig. 2, Table 3)

Traditional molecular tools, including quantitative RT-PCR, Northern blot and in situ hybridization, allow for examination of mucin expression at the RNA level [110–112, 116]. These methods are relatively popular due to their low cost, high specificity, and quantitative nature. A limitation of these types of approaches is that mRNA expression may not reflect protein levels. Further, no direct information regarding mucus/mucin secretion and/or mucus composition is provided. Luciferase reporter assays and ChIP assays can also allow for examination of pathways involved in mucin gene regulation [111, 113]. More recently, newer technologies, including single RNA-sequencing [12], have provided new information regarding mucin expression and properties of mucus-secreting cells. We expect that expanded use of this technology will continue to enhance our knowledge of mucus and mucins in both health and disease.

**Table 3:** Advantages and limitations of molecular and genetic methods and techniques used in mucus research

Method	Advantages	Limitations
Quantitative RT-PCR [110–112]	- Very specific quantitative information on mucin expression at the mRNA level - Inexpensive and easily applicable to most samples.	- Inability to detect increase in secretion. - Post-transcriptional modifications are also not detected.
Northern-blot (RNA-blot) assay [110, 111]	- Alternative method for detection of RNA. - Allows for separation of RNA molecules by size. - Provides information on number, length, and relative abundance of mRNAs expressed by a single gene	- More laborious, time-consuming and not as sensitive as qRT-PCR. - Requires large amount of tissue/sample, and high purity and quality of non-degraded RNA, which can be difficult for the large RNA molecules of mucins.
Luciferase reporter and Chromatin immunoprecipitation (ChIP) assay (promoter-binding) [111, 113]	- Luciferase reporter assay is commonly used to study gene expression at the transcriptional level. - ChIP allows for the specific study of molecular regulation and induction of mucin expression under various conditions.	- Most applicable in cell cultures. - Does not give quantitative information on mucin expression or secretion.
Using transgenic or knockout animals [89, 114, 115]	- Unique and valuable information on the overall function and/or effects of overexpression/depletion of each mucin throughout the lifespan of an animal model. - Can be used for determination and verification of mucin-regulation pathways.	- Most often applied in rodents. - Often have to be used in tandem with other techniques to verify the effect. - Depending on the model species, can be expensive, time-consuming.

Major breakthroughs in the field of mucus biology occurred when genetically-modified rodent models that overexpressed or lacked specific mucins were generated [89, 114, 115]. Muc5B knockout mice have shown the importance of muc5B in MCT and inflammatory cell responses (e.g., phagocytosis and apoptotic cell clearance), whereas muc5AC knockout models did not show any significant MCT deficiencies [114]. However, muc5AC knockout mice had a 74% reduction in airway obstruction when challenged with antigens to mimic allergic asthma. These results were observed without a dampening of inflammatory responses [115], suggesting that muc5AC was the major contributor to inflammation-induced airway obstruction. Muc5AC overexpressing animals, on the other hand, have been instrumental in showing the importance of muc5AC in the protection of the airways against viral invasions [89]. Specifically, an approximately 18-fold increase in muc5AC expression did not alter MCT, but instead protected the animals from aerosolized influenza challenge [89]. Thus, these animal models have provided unique insight into the specific roles of muc5AC and muc5B in airway health and disease. They also provide a unique advantage in that mucin biology and function can be studied with all the critical in vivo regulatory systems intact. Given species differences in airway structure and physiology, extrapolations from rodents to humans still requires caution. As such, additional comparative studies using other species might be beneficial.

### Quantitative and semi-quantitative protein detection of mucus and mucin (Fig. 2, Table 4)

Standard protein techniques, such as ELISA [117, 118] and antibody detection-based western blotting [100, 119], are frequently used to study mucus and mucins. These techniques are appealing because of their direct measurement of mucin and because of their quantitative nature. Further, the equipment required to do these types of experiments is fairly standard. Some limitations include the laborious methods required to isolate mucin (highlighted below), as well as the potential to not obtain an adequate sample volume. There are also specific technical considerations for ELISA procedures that are contingent upon the method used. For example, an absorption ELISA requires that the antigen be immobilized to a plastic surface and detected with an antibody, whereas a sandwich ELISA captures the antigen using an antibody-coated surface and then detects the antigen using another antibody [121, 122]. Although the absorption ELISA is simpler and less-time consuming, mucins absorption to a plastic surface requires a modified surface, which then enables the absorption of reduced mucin proteins [65, 123]. On the other hand, the sandwich ELISA offers greater sensitivity and specificity but is more time-consuming and laborious [122]. Additional approaches for examining mucus and mucins at the protein level include measuring percent solids [13, 88], dot-blot assays [120] and proteomics [124–126]. Although percent solids and dot-blot assays are simpler, they are less informative and less sensitive. Proteomics, on the other hand, is highly sensitive and a powerful tool to interrogate mucus. It can provide information regarding the relative abundance and types of proteins present in a mucus sample [127]. Proteomics also provides information regarding biochemical properties of mucins, as highlighted below.

**Table 4:** Advantages and limitations of quantitative and semi-quantitative protein detection methods for mucus/mucin measurement

Method	Advantages	Limitations
Percent solid matter [13, 88]	- Used for quantitative determination of mucus viscosity and water/solids ratio by measurement of the decrease in weight of mucus samples after oven drying. - Simple and inexpensive.	- Not an exact measurement of mucin, as the percent dry matter may increase/decrease due to changes in non-mucin molecules (e.g. inflammatory-cell derived products).
ELISA [117, 118]	- Simple and relatively sensitive detection/quantitation of proteins in liquid samples. - Can be used for in vivo collected sputum and ASL.	- Antibody needs to be specific for mucin of interest and epitope should avoid homologous regions/repeats between mucins. - A purified species-specific mucin standard should be used, which is not always available. -
SDS-PAGE/western blot assay [100, 119]	- Inexpensive and relatively accurate measurement of specific proteins in liquid samples and tissue homogenates. - Can be used together with housekeeping molecules for proper quantitation. - Allows for the detection of normal and modified forms of the same protein (after stripping of initial labeling)	- Antibody needs to be specific for mucin of interest and epitope should avoid homologous regions/repeats between mucins. - Requires denaturation of mucins for running on SDS-PAGE gels or agarose gels for proper separation of the larger molecules.
Dot-blot (Slot-blot) assays [120]	- Inexpensive and quick alternative to western blots for antibody comparison and assessment in a large number of samples.	- Does not separate proteins by size. - Not as sensitive as western blot (quantification is based on intensity image analysis of dots). - Does not typically utilize housekeeping proteins to normalize the signal intensity.

### Measurement of biophysical/biochemical properties (Fig. 2, Table 5)

The biophysical and biochemical properties of mucus are most affected by its composition, and greatly impact the ability of mucus to be cleared out of the airway. Thus, there is great interest in understanding the biophysical and biochemical properties of mucus and mucin. Historically, studying mucin requires rigorous and tedious isolation, solubilization and purification procedures [128, 129, 138–140]. Early in vitro studies relied predominantly on cesium bromide density gradients and sephadex chromatography, alone or coupled with proteolytic digestion and/or denaturing agents [128–130], which provides information about its biochemical properties and structure. An advantage of chromatography is that it provides valuable information on mucin molecular charge and size [128, 129, 136]. Further, through additional processing using traditional (e.g., Edman degradation [141]) or more advanced techniques (e.g., mass-spectrometry [142, 143]), glycosylation patterns of mucins can be resolved [13]. This is important because glycosylation patterns greatly impact the biophysical nature of mucins, as the O-glycosylation of the protein backbones has been shown to induce higher molecular rigidity and extended conformation [15, 27]. This change in rigidity is due to the polyanionic and dielectric properties of the sulfated and sialic sugars, especially when introduced repeatedly in the heavily glycosylated tandem repeat regions of mucins [27]. Additionally, the heavy glycosylation of mucins has been linked to increased hydration of the molecules, giving them their gel-like properties, and are critical in establishing their rheological properties (from flexible to rigid and brittle) [27, 144].

**Table 5:** Advantages and limitations of methods and techniques used for measuring biochemical and biophysical mucus properties

Method	Advantages	Limitations
Isolation, fractionation and purification of mucins [128–130]	- Required steps for all initial molecular/biochemical characterizations.	- Labor intensive and may be expensive. - Loss of sample during the process, and therefore cannot be used solely for quantification purposes. - Care must be taken that the mucins do not become degraded.
Glycosylation analysis [17] and mass spectrometry [127].	- Provides valuable information on species- and organ-specific glycosylation and post-transcriptional modifications of mucins. - Precise qualitative and quantitative information on different molecules in the sample, most often proteins and carbohydrates.	- Expensive system and materials. - Identification of proteins/molecules require protein libraries for each animal species of interest and knowledge about glycosylation sites.
Viscoelasticity of mucus (laser/light scattering analysis [131], direct rheometry [132], and fluorescence recovery after photobleaching (FRAP) [13, 133–135])	- Laser scattering or quasi-elastic (dynamic) scattering is used specifically for molecular size distribution and for mucin conformation and chain dimensions analysis. - FRAP assay is easily applicable for in vitro/ex vivo studies. - Viscosity/elasticity under shear stress conditions can be done directly in rheometer machines but requires higher amounts of sample.	- Use mathematical modeling equations to calculate the viscoelasticity, which can introduce errors if not performed or calculated correctly. - They do not give information on the quantity or specificity of single mucin component. - FRAP and other microrheology techniques depend on the diameter and non-adhesiveness of labelled particles used.
Chromatography separation and detection [128, 129, 136]	- specific technique for separation and molecular analysis of biological substances. - Provides information on molecular charge and size.	- Chromatography is expensive and labor-intensive. - If radioactive detection use, handling and disposal, is expensive and environmentally unfriendly.
Metabolic labeling/ Radiolabel discharge measurement or autoradiography [129, 137].	- Can be used to measure amount of secreted radioactive isotope-labelled substance (e.g. ^3^H-D-glucoseamine, or iodo[−^14^C]acetamide) incorporated easily in the newly produced mucins. - Historically used for characterization of mucin size in chromatographically separated fractions and for quantitation of total mucus secretion after treatments (as ratio of radioactivity detected at baseline and after treatment).	- Requires radioactive substance handling and exposure. - Materials are strictly regulated and expensive to dispose of. - Not very sensitive to specific mucin secretion. - Applicable only in vitro/ex vivo and on sputum samples from patients.

Radiolabeling (metabolic labeling) of mucins also provides information regarding the total release of glycoproteins, as well as the functional state of the secreting cells [128, 129, 137, 140, 145, 146]. When radio-labeled monosaccharides (such as fucose) are used, they can also additional more specific information regarding glycosylation [129, 137]. Although, radio-isotope incorporating techniques were essential in initial characterization studies of mucins, they are growing less popular.

Most early mucin characterization studies have focused on the physical and rheological properties of purified mucins (molecular weight, elasticity, light scattering and sedimentation velocity), as well as on the biochemical properties (pH, amino-acid and monosaccharide-moiety composition) [17, 130, 132, 138, 147, 148]. The rheological properties of mucins are among the most important determinants for proper MCT [27, 148], and they are highly dependent not only on the relative ratios of the different mucins and their biochemical features, but also on the properties of the solvent and other proteins or salts. Together, these molecules interact with mucins to form the mesh structure of the mucus gel layer [27, 148]. The normal properties of mucus are those of a viscous liquid under high shear rates, and of an elastic solid at low shear rates [148]. Classical direct rheometry techniques, make use of special instruments like the cone-and-plate rheometer, the capillary viscometer, or the filancemeter, which rely on the subjection of collected mucus samples to known forces like shear rate, torque, strain or traction [132, 148]. As a result, they enable the measurement and calculation of viscosity and elasticity under different conditions [148]. Light or laser scattering techniques utilize frequency shift and multiangle scattering of a light (laser) beam from moving molecules in samples, and the collected data gives information of the absolute molar mass and the average size of mucins in solution [100, 130, 147]. It has also been shown to be useful in the assessment of the motility of respiratory cilia [131].

The aforementioned methods described the macrorheology of mucus. However, the microrheological properties of the mucus gel layer are also of interest, and can provide significant information on the mesh-pore size and thus penetrability of infectious, toxic or other agents into the gel, as well as the mucus viscoelasticity [148]. Visually-based techniques like the fluorescence recovery after photobleaching (FRAP) assay are used to determine the viscosity of mucus by measuring the two-dimensional lateral diffusion of a thin film of a FITC labelled dextran powder (or other fluorescent molecules) applied on the mucus surface, and its fluorescence recovery time after light exposure [13, 133–135]. This technique is especially applicable in ciliated epithelial cultures or tracheal explants [13, 133–135] or on freshly collected mucus samples [13]. Similar to the other visual techniques described earlier, factors like molecular/particle size and adhesiveness, pH-sensitivity, photostability and brightness [148] need to be accounted for when using FRAP. Further, careful consideration of the mathematical models used to calculate the rheological properties is necessary for proper and rigorous evaluation [149].

## Conclusions

The heterogeneous nature of mucus glycoproteins, their large molecular sizes and complex intermolecular interactions, continues to make studies aimed at elucidating mucin production, secretion, abundance, and biophysical properties, challenging [49–51, 148]. Despite these challenges, there are numerous techniques available to study mucus and mucin, each with distinct advantages and limitations.

For example, purification methods are laborious while radiolabeling methods are expensive and pose health and environmental risks. Thus, these techniques are generally in decline and would benefit from a refined methodology. ELISA methods based on antibody detection remain a relatively inexpensive method to detect mucin concentrations in patient sputum, BAL and other body fluids [117, 118], but require mucin-specific antibodies (e.g., MUC5AC vs. MUC5B). Western blot methods are also useful for detection of non-gel-forming mucins in biological fluids and tissues [100, 119], but can require labor intensive solubilization and purification to allow for adequate separation during gel-electrophoresis. Proper detection in western blots also depends on the availability of species-specific mucin-labeling antibodies.

Histological and immunocytochemical imaging methods remain gold standards for detection of goblet cell hyperplasia/metaplasia, mucus plugging, localization of non-secreted mucins or co-localization of mucins with other molecules in vitro, ex vivo or post mortem [90, 92, 93, 95, 100], but may be more difficult to utilize in vivo [91] or in patients [20]. They are also limited in that they provide episodic information about a specific point in time. Thus, there has been a push to develop imaging methods that can be implemented in vivo that allow for capturing of dynamic information. Consistent with that, several advances in imaging techniques, including X-ray synchrotron [96] and quazi-monochromatic X-ray phase-contrast imaging [1], have been applied successfully to measure MCT and mucus plugs in vivo.

Molecular and genetic methods, and particularly RNA extraction with RT-PCR, have also become increasingly utilized for the detection of changes in expression levels of mucins [110–112]. They are also useful for the detection of downstream and upstream mechanisms in the regulation of mucin expression/secretion [111, 113]. As mentioned, genetically engineered rodent models have provided unprecedented insight into the role of Muc5AC and Muc5B in airway health and disease [89, 114, 115]. Because of species differences in mucin and airway physiology and anatomy [51], it is worthwhile and recommended that additional animal models be explored.

The biophysical and biochemical properties of mucus can be assessed using many different approaches. Although separation chromatography was used frequently during many of the initial glycobiology studies [128–130, 136], pairing chromatography with more recent technology, such as mass spectrometry, has become increasingly popular [13, 127]. Additionally, the gold standard for measuring viscoelastic properties of mucus is direct rheometry, but these methods typically require a significant volume of mucus and are limited to in vitro studies [17, 148]. Thus, FRAP as a tool to measure viscoelastic properties has become an effective and valuable approach that has fewer volume limitations [13, 133–135, 148]. The development of a nanoprobe to measure the biophysical properties of mucus in vivo in a somewhat non-invasive manner could significantly enhance the field. For example, the fabrication of flagellated nanobots inspired by bacteria has been reported [150, 151]. Thus, it is possible that flagellated nanobots or some iteration of flagellated nanobots might offer new approach to measure the viscoelastic properties of mucus and mucins.

Multiple strategies are required to study mucus and mucins. The strength of using a multi-strategy approach is the reduction in errors that are likely to occur by using a single technique. Unfortunately, many aspects of mucin regulation are still unclear, especially on the scale of longitudinal changes in mucus properties within the same subject or patient. Thus, though great leaps in technology over the past few decades have been made, there is still a need for the development of fast and easy method(s) for detection and quantitation of mucins and mucus, especially as it relates to clinical samples and in patients.

## Data Availability

All data generated or analyzed during this study are included in this published article.

## Abbreviations

AB/PAS: Alcian Blue
AREST CF: Australian Respiratory Early Surveillance Team for Cystic Fibrosis
ASL: Airway surface liquid
ChIP: Chromatin immunoprecipitation
COPD: Chronic obstructive pulmonary disease
e.g.: Exempli gratia (for example)
ELISA: Enzyme-linked Immunosorbent assay
FEV1: Forced expiratory volume in 1 s
FITC: Fluorescein isothiocyanate
FRAP: Fluorescence recovery after photobleaching
MCT: Mucociliary transport
mRNA: Messenger ribonucleic acid
MUC: Human mucin (denoted with the number after MUC)
muc5AC: Mucin 5 AC
muc5B: Mucin 5B
NHLBI: National Heart, Lung, and Blood Institute
PAS: Periodic Acid-Schiff stain
PCL: Periciliary layer
RT-PCR: Reverse transcription polymerase chain reaction
SDS-PAGE: Sodium dodecyl sulfate polyacrylamide gel electrophoresis
SPIROMICS: Subpopulations and Intermediate Outcome Measures in COPD Study
